# Long-Term Electromyographic Monitoring of the Stapedius Reflex via Implanted Electrodes in Sheep: Toward Objective Autonomous Cochlear Implant Fitting

**DOI:** 10.3390/s26134224

**Published:** 2026-07-03

**Authors:** Dirk Arnold, Jose Luis Vargas Luna, Orlando Guntinas-Lichius, Gerd Fabian Volk

**Affiliations:** 1 Department of Otorhinolaryngology, Jena University Hospital, 07747 Jena, Germany; orlando.guntinas@med.uni-jena.de (O.G.-L.); fabian.volk@med.uni-jena.de (G.F.V.); 2 MED-EL Medical Electronics, 6020 Innsbruck, Austria; jose.vargas@medel.com; 3 Facial-Nerve-Center Jena, Jena University Hospital, 07747 Jena, Germany; 4 Center for Rare Diseases, Jena University Hospital, 07747 Jena, Germany

**Keywords:** stapedius, reflex, electromyography, EMG, sheep, chronic, acoustic, stimulation, middle ear admittance

## Abstract

**Highlights:**

Long-term recordings of myoelectrical activity of the stapedius muscle as a detection method for the maximal comfortable loudness level are possible.

**What are the main findings?**

- The stapedius reflex remains physiologically elicitable and measurable using EMG electrodes even after long-term implantation, provided that the electrodes have been placed on the surface of the muscle and their integrity was maintained.
- Correct placement of the EMG electrodes is important to detect myoelectrical signals.

**What are the implications of the main findings?**

- EMG measurements could be implemented in cochlear implants for autonomous fitting.
- Random placement of electrodes will not allow for reliable EMG measurements.

**Abstract:**

Objective fitting measures offer a means to circumvent the subjectivity of cochlear implant programming, with the stapedius reflex representing one robust predictor of the maximum comfortable loudness level. With the present study, it was investigated whether long-term electromyographic measurements of the stapedius muscle using implanted electrodes are feasible. In nine sheep, myoelectrical activities were recorded intraoperatively and synchronized with middle ear admittance as a reference signal. For acoustic stimulation pure tones with different frequencies were used. The electrodes were placed at the stapedius muscle surface after exposing it via the retrofacial approach. EMG-based detection of the stapedius reflex was achievable over six months when electrode integrity and placement were preserved. The treated muscles were subsequently excised, cut and examined histologically. No signs of atrophy were found in the muscles examined. However, the histological section series showed a clear division of the muscle from proximal to distal, the ratio between tendon and muscle fibers being most pronounced in favor of the muscle fibers in the proximal section. The integration of an electromyography-based measurement method for the objective determination of the stapedius reflex threshold and thus, for the long-term adjustment of cochlear implants, appears possible and could potentially enable autonomous fitting of implants.

## 1. Introduction

Cochlear implants (CIs) have evolved remarkably since the first single-channel device was implanted in 1957 by Djourno and Eyriès, progressing to modern systems with up to 26 electrodes and smartphone-based control interfaces. These advances have enabled many CI users to achieve high levels of speech recognition. After the development of the first multi-channel electrode arrays the possibility of decoupling CI fitting from subjective patient feedback was discussed and the search for objective fitting strategies began [1,2].

Despite extensive research into objective fitting methods—including electrically evoked auditory brainstem responses (EABR), electrically evoked compound action potentials (ECAP)/neural response telemetry (NRT), and electrically evoked stapedius reflex thresholds (eSRT)—routine CI fitting still relies heavily on psychoacoustic methods [3,4,5,6,7,8,9,10,11]. These methods are inherently limited in populations that are unable to provide reliable feedback, such as infants or individuals with cognitive impairment, and in patients with no prior auditory experience where adequate perception of new sensory impressions is difficult. Moreover, subjective assessments are time-consuming and prone to misjudgment, often resulting in suboptimal stimulation levels and reduced speech recognition outcomes [11,12,13].

Objective fitting methods are essential for optimizing speech recognition outcomes in cochlear implant users [7,14,15]. Among these approaches, the eSRT has consistently demonstrated high reliability for estimating the maximum comfortable loudness level (MCL) [1,5,6,11,13,14,16,17,18,19,20,21,22,23,24]. The stapedius reflex—a protective muscular response to intense acoustic stimulation—can be assessed either indirectly via middle ear admittance measurements or directly using electromyography (EMG) [9,25,26,27,28,29]. However, clinical eSRT assessment based on middle ear admittance is frequently compromised by factors such as middle ear effusion, tympanic membrane condition, probe placement variability, and limited patient cooperation. These factors reduce measurement accuracy and reproducibility [30,31] but are intrinsic to admittance-based techniques and do not affect EMG-based monitoring of stapedius muscle activity.

The long-term stability of SRT after CI implantation remains a subject of debate. While also very recent studies report adaptive changes in SRT and MCL over time [7,12,13,23,32,33,34,35,36,37,38,39,40,41], others, such as Pitt et al. (2021), found no significant longitudinal variation in SRT across a large cohort [42]. Conversely, Brotherton et al. (2017) demonstrated a significantly lower SRT following a 4-week sealing of one ear and its subsequent normalization [38]. Raghunandhan et al. (2014) reported a postoperative increase in eSRT and MCL, attributed to tissue remodeling around the CI electrode up to 1 year post-implantation [13]. In addition, Muigg et al. (2025) demonstrated SRT changes within the first 4 years after CI implantation [41]. These findings suggest that SRT may be influenced by biological and procedural factors, including neuronal adaption, healing dynamics and electrode positioning.

Given the importance of accurate and repeatable fitting, frequent post-implantation assessments are essential. Even minor deviations in mapping can significantly impact speech recognition [7,43,44]. Integrating EMG electrodes into CI systems could enable routine, objective SRT measurements by audiologists and speech therapists. Recent technological advances even lead to expect the feasibility of autonomous CI fitting based on real-time EMG feedback.

In this study, we evaluated whether the stapedius reflex remains elicitable following long-term implantation, focusing on the preservation of stapedius muscle integrity and electrophysiological responsiveness. Using chronically implanted custom-made EMG electrodes in an ovine model, selected for its anatomical similarity to the human temporal bone [45], we conducted a six-month longitudinal assessment. By combining electrophysiological measurements with post-mortem histological analysis, this work aims to establish whether chronic implantation alters the functional viability of the stapedius reflex, thereby providing a physiological basis for future EMG-based stapedius reflex threshold (SRT) measurements in cochlear implant applications.

## 2. Materials and Methods

### 2.1. Ethical Approval and Animal Welfare

All procedures were conducted in compliance with European and German animal welfare regulations. The study protocol was approved by the Committee for Animal Research of the State of Thuringia, Germany (approval code: UKJ-19-006). Animal husbandry and all procedures were carried out at the Animal Facility of the Jena University Hospital.

### 2.2. Animals and Study Design

Nine adult female Merino sheep (age: 4–5 years; weight: 70–100 kg) were included in the long-term study, and one adult female Merino sheep cadaver used exclusively for histological analysis. Upon arrival, animals underwent a minimum acclimatization period of five weeks to allow for health screening and intensive handling by trained personnel, which significantly facilitated postoperative care. Animals were included in the study only if the presence of a stapedius reflex was confirmed prior to the implantation procedure.

**Figure 1:**
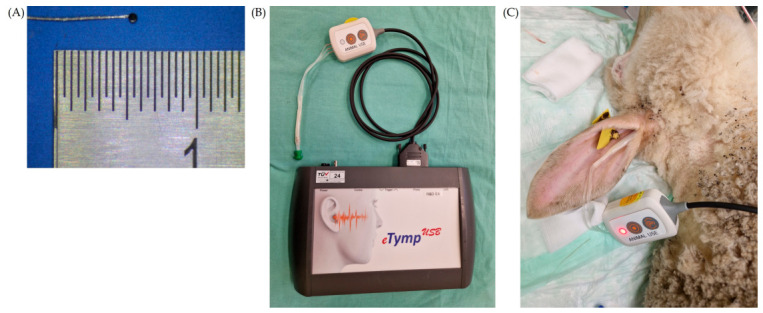
(**A**) Custom-designed platinum–iridium EMG disk electrode, comprising a stranded wire (7 single wires, 10IR9.49T) with a tip of approximately 0.65 mm diameter. (**B**) Adapted scientific middle ear admittance meter (eTymp by Biomed Jena GmbH, Jena, Germany). (**C**) Pre-screening for stapedius reflex before surgery with eTymp and probe elongation.

The left stapedius muscle was accessed via a retrofacial approach [46,47]. Two custom-designed platinum–iridium EMG disk electrodes, comprising a stranded wire (7 single wires, 10IR9.49T, Sigmund Cohn Corp., Mt Vernon, NY, USA) with a disk tip of approximately 0.65 mm diameter (Figure 1A), were implanted on the surface of the muscle. Recordings were scheduled at four time points: during implantation (baseline), and at 1, 3, and 6 months post-implantation. The experimental configuration was designed as a proof of concept to test the underlying physiological hypothesis, rather than as a clinically deployable system, and therefore prioritized experimental access over long-term mechanical robustness.

Following the final measurement, both stapedii (left and right) were surgically excised and subjected to histological examination to assess tissue integrity and potential implant-related changes.

### 2.3. Surgery Preparation and Pre-Screening

The SR monitoring via middle ear admittance was performed using a commercial tympanometer device (eTymp, Biomed Jena GmbH, Jena, Germany) (Figure 1B,C). Similar to Pohl et al. 2017, an elongated probe tip was implemented to accommodate anatomical differences between the human and ovine external auditory canal [48]; however, in our design, the elongation preserved the separation of the three tympanometer channels (pressure delivery, acoustic stimulation, and microphone) up to the distal end. In addition, the outer ear was cleaned and let to dry in the stable one day before the surgery.

The sheep were sedated and prepared in the stables for pre-screening with short-acting midazolam (0.1–0.3 mg/kg, Midazolam-Ratiopharm^®^ 15 mg/^3^ mL, Ratiopharm, Ulm, Germany) and ketamine i.m. (10–20 mg/kg, Ketamin 100 mg/mL, WDT, Garbsen, Germany). The neck and the skin around the ears were shaved with a long hair trimmer (Aesculap^®^ Vega GT 606, Aesculap, Tuttlingen, Germany) before a Safety IV Catheter with injection port (Vasofix^®^ Safety, B. Braun, Melsungen, Germany) was placed into the jugular vein. A syringe driver was connected to apply propofol (18–40 mg/kg/h, bolus 2 mL/kg BW, Narcofol^®^, CP-Pharma, Burgdorf, Germany). The relaxed animals were intubated with a tracheal tube of 8.5 mm or 9 mm (TrachealTube, Smiths Medical Portex^®^, Minneapolis, MN, USA) and ventilated with isoflurane (1–3.5 Vol%, Isofluran CP^®^ 1 mL/mL; CP-Pharma, Burgdorf, Germany). Fentanyl (i.v. 2 µg/kg BW, Fentadon^®^ 50 µg/mL, Dechra, Aulendorf, Germany) was used for analgesia as bolus. Immediately after induction of anesthesia on the operation table, the presence of the stapedius reflex on each animal was determined. Before the reflex test, the ears were cleaned with cotton buds (Meditrade^®^, Kiefersfelden, Germany) to remove any remaining earwax to reduce the risk of probe contamination or clogging.

### 2.4. Electrode Implantation

Immediately after induction of anesthesia, the presence of an acoustically evoked stapedius reflex was reassessed. From the nine animals included, one did not present any reflexes during this pre-screening and was, therefore, excluded from the study and recovered without implantation. The other eight animals exhibited a reliable reflex and were subsequently implanted. All implantations were performed on the left side to facilitate intraoperative rumen venting via a cannula, thereby preventing abdominal distention during prolonged anesthesia.

After skin incision caudoventrally of the ear, the tendon of the sternocleidomastoid muscle was partially removed to expose the basis of the mastoid bone. A hole was drilled (UNIDRIVE^®^ II-plus, Karl Storz, Tuttlingen, Germany) to find the facial nerve, which was identified using brief stimulation pulses (3 Hz and 1.0 to 2.5 mA) via a bipolar nerve locator connected to a NeuroSign 100 (MAGSTIM Company Limited, Whitland, WLS, UK). The acoustic control was based on the myoelectrical activity of the facial muscles, which were monitored using 3 subdermal needles (two for bipolar recording + one grounded), placed on the left cheek.

**Figure 2:**
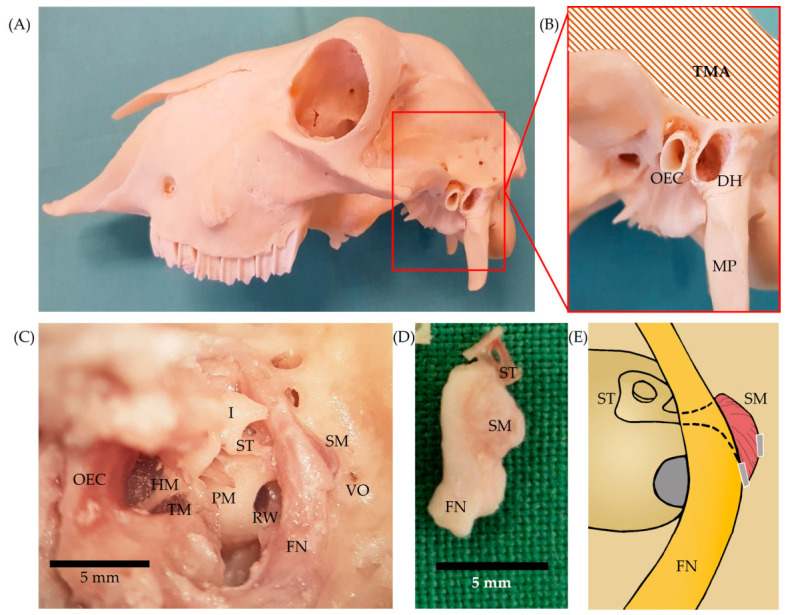
Sheep anatomy and electrode placement: (**A**) sheep skull with artificial hole for retrofacial approach; (**B**) close-up of sheep skull; TMA = area of the temporalis muscle; OEC = outer ear channel; DH = drilled hole for retrofacial approach; MP = mastoid process; (**C**) dissected middle ear of a sheep cadaver, I = incus, ST = stapes, SM = stapedius muscle, OEC = outer ear channel, HM = handle of the malleus, TM = tympanic membrane, PM = promontory, VO = vestibular organ, RW = round window, FN = facial nerve; (**D**) dissected stapedius muscle with connected stapes and facial nerve; (**E**) schematic drawing with electrode positions (white-rimmed discs located on the muscle belly).

The facial nerve was then traced in depth to reach the stapedius muscle by the retrofacial approach without opening the middle ear, allowing for ipsilateral acoustic stimulation (Figure 2A,B). It is imperative to avoid injury to the facial nerve and the labyrinth structures, which are situated close to the stapedius muscle in sheep (Figure 2C,D). In addition, with permanent irrigation, drilling was carried out at low speeds in the vicinity of the facial nerve to prevent damage due to overheating. The stapedius muscle was carefully exposed caudally to the facial nerve (Figure 2C), and the electrodes were placed at its surface (Figure 2E).

For electrode placement, a pocket between the stapedius muscle and the petrosal bone was prepared by separating the origin of some muscle fibers. The second electrode was placed at the opposite side between the facial nerve and the muscle belly as far away from the other electrode as possible (Figure 2E). The electrodes were fixed in position with fibrin glue (Surgibond, SMI AG, St. Vith, Belgium). A part of the electrode cable was placed within the drilled hole, which was finally closed using bone cement. The larger part of the lead was tunneled to the neck, where the connector was positioned in a pre-prepared subcutaneous pocket. After complete fixation, the reflex was monitored again by the middle ear admittance and by EMG recording. After several repetitions, the EMG system was disconnected and the wound was rinsed with ampicillin (Ampi-Dry, Veyx, Schwarzenborn, Germany) before the skin incisions were closed (Vicryl^TM^ 3/0 and Prolene^TM^ 2/0; Ethicon^®^, Norderstedt, Germany) and carprofen (1–4 mg/kg BW i.v., Carprosol^®^ 50 mg/mL, CP-Pharma, Burgdorf, Germany) was given. Bandages were applied around the ears and the neck (Inadine^®^, Solventum, Kamen, Germany, Artiflex^®^ Soft, BSN medical, Hamburg, Germany, Elastomull^®^ haft hospital, Leukoplast^®^, BSN medical, Hamburg, Germany, tg^®^fix, Lohmann & Rauscher, Rengsdorf, Germany) after connecting an elongation to the Safety IV Catheter. Then the animal woke up. The sheep were monitored over the next few days. The bandages were reapplied daily, and the catheter was used to apply analgesia (carprofen 1–4 mg/kg BW, i.v. Carprosol^®^ 50 mg/mL, CP-Pharma, Burgdorf, Germany) and antibiotics (enrofloxacin 5 mg/kg, i.v., once daily for 3 days, Baytril^®^ 50 mg/mL, Bayer, Leverkusen, Germany) for 4 days postoperatively. After 4 days, long-term antibiotics (oxytetracycline 20 mg/kg BW, i.m. Terramycin^®^ LA 200 mg/mL, Zoetis, Berlin, Germany) were administered. To prevent a painful reaction to the oxytetracycline, lidocaine (1 mL/cm injection site, Lidor^®^ 20 mg/mL, WDT, Garbsen, Germany) was used.

### 2.5. Follow up and Final Control

First, the connector was exposed and the impedance between the electrodes was measured to verify the integrity of the cables. Then, bipolar EMG measurements were performed during ipsi- and/or contralateral acoustic stimulation. In some cases, alternative monopolar measurements were performed. If impedance exceeded expected values and cable breakage was suspected, access to the electrode lead proximal to the breakage site was attempted only during the final (6-month) measurement. If no reflex was recognizable with middle ear admittance or in the EMG signal, the measurements were repeated after the anesthesia was lowered as much as possible without compromising the animal’s health.

A flowchart of the testing, follow-up intervals and histological investigations is depicted in Table 1.

At the end of the final measurements the animals were euthanized in deep anesthesia using pentobarbital sodium (100–150 mg/kg BW, i.v., Euthadorm^®^, 500 mg/mL, CP-Pharma, Burgdorf, Germany). Afterwards, the electrode positions were verified, and the stapedius muscle of the left and the right side were dissected for further histological analysis (Figure 2D).

### 2.6. Histological Treatment

The left and right stapedii of all implanted sheep (*n* = 8) and one additional control (muscles of a cadaver) were fixated in 4% formalin (Formaldehyd, Otto Fischar, Saarbrücken Germany), decalcified with Osteomoll^®^ (Sigma Aldrich, Darmstadt, Germany) and subsequently classically histologically treated. As the electrodes were always implanted on the left side, the right stapedius muscle served as control (Table 1).

The prepared muscles were embedded in paraffin (Sigma-Aldrich, Taufkirchen, Germany) before 7 µm thick serial sections were made using a microtome (HM 360 Microm, Marshall Scientific, Hampton, NH, USA). The slices (approx. 300 per muscle) were mounted on microscope slides and stained with Heidenhain’s AZAN trichrome stain to discriminate muscle and connective tissue.

All slices were photographed using an Axioscan Z1 (ZEISS, Oberkochen, Germany) and visually analyzed by three histologically skilled examiners. One of them was familiar with the experimental design and procedures, while the other two assessed the histological sections in a blinded manner. They were informed only that the specimens were muscles implanted with electrodes for signal recording and that they had been excised at the end of a long-term study for histological examination. The evaluation focused on identifying differences between the left and right muscle bellies, specifically the presence of increased muscle fiber atrophy, connective tissue, fibrotic or scar tissue, or conspicuous fat accumulations.

### 2.7. Outcome Measures

#### 2.7.1. Acoustic Reflex

Acoustic stimulation was delivered using eAudio software (BioMed Jena, Jena, Germany), whereby one to three repetitions of pure-tone stimuli at frequencies of 500 Hz, 1000 Hz, 2000 Hz, and 4000 Hz was applied. Stimulus intensities were variable but always within 70–110 dB SPL ((sound pressure level) maximum 100 dB SPL for 4000 Hz), with a fixed duration of 0.5 s and a post-stimulus observation window of 1 s. One recording consisted of an intensity and frequency sweep. Intraoperatively, if no response was observed, probe repositioning was attempted or anesthesia was reduced to a minimum, and after a few minutes the test was repeated.

To synchronize EMG recordings with acoustic stimulation, a trigger signal from the tympanometer was integrated into the EMG acquisition system. Reflex responses were classified into two categories based on amplitude, waveform morphology, and reproducibility across repetitions: “Reflex” and “No Reflex” (Table 1).

#### 2.7.2. Electromyography (EMG)

Myoelectrical activity was detected from the stapedius muscle via a pair of custom-made platinum–iridium disc electrodes (MED-EL, Innsbruck, Austria) (Figure 1A). A subcutaneous needle electrode was inserted as a reference. The bipolar EMG was conditioned with an EMG front-end (Dual Bio-Amp, AD Instruments, Dunedin, New Zealand) and recorded at 100 kHz with a PowerLab (AD Instruments, Dunedin, New Zealand). The front-end was configured to bandpass filter from 1–5000 Hz.

During the recording, electromagnetic noise was kept to the minimum by disconnecting the operation table, electrosurgical unit, and driller from the power outlets. Cables were shielded either with external shielding tubes or shielded cables were used, if the noise was still high, the 50 Hz notch filter from the EMG front end was activated.

### 2.8. Signal Processing

The middle ear impedance and EMG signals were integrated in Matlab 2023a (The Mathworks Inc., Natick, MA, USA) for post-processing. Both signals were resampled to 10 kHz.

The middle ear impedance data were used as provided by the eAudio USB 2.0 software without further filtering. The data were aligned with the EMG using the trigger signal. To quantify the stapedius reflex response, the absolute value of the signal was computed, and the 90th percentile of the waveform within the first 200 ms following stimulus onset was extracted. This percentile-based metric was selected to approximate the peak response while reducing sensitivity to transient noise artifacts. A reflex was classified as present when the signal exceeded 0.03 mL, corresponding to the standard detection threshold implemented in the eAudio USB 2.0 software.

The middle ear admittance approach was selected based on prior work demonstrating the feasibility of tympanometry measurements in sheep [48], as both classical tympanometry and stapedius reflex detection via middle ear admittance rely on similar measurement principles. However, probe placement in sheep was frequently suboptimal due to the experimental setup constraints, e.g., sheep position, dampness in the outer ear canal and time limitations during surgical procedures. This often resulted in noisy recordings, underestimated reflex amplitudes, or absent detectable responses, even in cases where reflex activity was confirmed by EMG or was consistent with responses observed at adjacent stimulation intensities.

To mitigate these limitations, a secondary visual review of the recordings was performed. Automatically detected reflexes were screened to exclude false positives caused by noise artifacts, while signals not classified as reflexes were re-evaluated for subthreshold but reproducible and physiologically consistent responses, including cases in which increasing stimulus intensity elicited a progressively stronger response. This classification approach was intended to preserve biologically plausible reflex detections while accounting for the known technical limitations of tympanometric measurements in this experimental setting.

Raw EMG signals were filtered using a 32nd-order IIR bandpass filter (80–800 Hz) to isolate stapedius muscle activity and suppress low-frequency artifacts and high-frequency noise. Persistent 50 Hz powerline interference and harmonics were removed using a pattern extraction filter based on 40-cycle modelling. Additional frequency-specific noise arose from unshielded cable sections and elevated impedance due to mechanical stress or unstable micrograbber connections. These vulnerabilities led to contamination from the tympanometry device probe tone (226 Hz) and stimulus (500 Hz), and additional cross-over from surgical equipment ranged between 500 and 550 Hz. These artifacts were selectively attenuated using 32nd-order IIR band-stop filters, each targeting ±10 Hz around the identified frequencies, or broader ranges when peaks clustered closely.

To determine the presence of a stapedius reflex in the EMG recordings, two quantitative features were extracted from each stimulus response. The primary metric was the maximum amplitude of the EMG envelope within a post-stimulus window of 30–130 ms. The envelope was computed using a moving root mean square (RMS) with a window length of 10 ms. The second metric was the RMS ratio, defined as the RMS value within the post-stimulus window (30–130 ms) divided by the RMS of a 200 ms pre-stimulus baseline. While the peak amplitude criterion ensured a minimum level of muscle activity consistent with a reflex response, the RMS ratio was used to detect relative increases in activity and reduce susceptibility to elevated baseline noise.

As, to our knowledge, no prior reports exist describing stapedius muscle EMG recordings in chronic implantation settings, these metrics and their corresponding thresholds were derived empirically from the observed data as part of an initial signal characterization rather than as predefined or externally validated criteria. A response was classified as a reflex if the RMS ratio exceeded 1.15 and the peak amplitude reached or exceeded 3 µV_RMS_.

To further account for variability and noise inherent to the experimental recordings, a secondary visual review was performed. This review was based on expected electrophysiological characteristics of the stapedius reflex, including response reproducibility and a consistent increase in amplitude with higher stimulus intensity. Responses initially classified as reflexes were screened to exclude transient noise artifacts, while signals not meeting threshold criteria were re-evaluated for subthreshold but reproducible and physiologically consistent activity patterns. This combined quantitative and qualitative approach was intended to identify biologically plausible reflex responses while accounting for the limitations of the recording conditions.

Given the hierarchical structure of the data (stimuli nested within animals and stimulation conditions), analyses were restricted to descriptive statistics, and no formal statistical comparisons were performed.

## 3. Results

### 3.1. Animals

Of the nine sheep initially enrolled, eight underwent the full protocol and one animal (S08) was excluded prior to implantation due to the absence of a stapedius reflex during pre-screening. From the eight implanted animals, S04 was categorized as sham-implanted control, because post-mortem analysis revealed that both EMG electrodes had been incorrectly positioned and were not in contact with the stapedius muscle. Therefore, only the histological and middle ear admittance data were valid, and EMG recordings were discarded and marked as “not measured” in Table 1.

Across the eight treated animals, a total of 27 measurement sessions were conducted, encompassing implantation, follow-up, and termination procedures. Across follow-up sessions, electrode and lead integrity progressively degraded, limiting the availability of bipolar EMG recordings at later time points. This resulted in the omission of the second follow-up session in all animals, as EMG recordings became unfeasible despite changes in acoustic middle ear admittance. Nevertheless, elicitable stapedius reflex responses were observed in animals with one or two electrodes preserved. During the final measurement sessions, when breakages were identified, access to the electrode was restored in all animals by drilling through the bone cement to expose the proximal end of the lead before damage.

In total, data from seven sheep (S03, S05, S06, S07, S09, S10, and S11) were included in the EMG analyses, while middle ear admittance reflex assessments were available from eight animals (S03, S04, S05, S06, S07, S09, S10, and S11). Histological data were analyzed from the seven implanted animals (S03, S05, S06, S07, S09, S10, S11), one sham-implanted control (S04) and one cadaveric control (see Table 1). The left stapedius muscle of S03 was lost during dissection, but all remaining samples were successfully preserved for histological evaluation.

During the implantation session, preoperative stapedius reflexes were detected via middle ear admittance in all eight animals, although intraoperative reflexes were not detectable in three cases (S05, S10, S11). EMG responses were observed in at least one measurement in four animals (S03, S06, S07, S09) out of seven animals with valid EMG datasets (S03, S05, S06, S07, S09, S10, S11).

During the follow-up sessions, intraoperative reflexes were detected via middle ear admittance in all but one animal (S10). However, EMG recordings were compromised in all cases due to lead breakages affecting one or both electrodes. In one animal (S03), reflex-related EMG activity was successfully recorded using a monopolar configuration.

Termination measurements showed consistency with the implantation results. The same three animals that lacked intraoperative reflexes during implantation (S05, S10, S11) also showed no reflexes at termination, and correspondingly, no EMG signals were detected. Middle ear admittance data show reflexes in the remaining five animals, including S04. Among these, all four animals with correctly placed electrodes exhibited EMG activity during acoustic stimulation.

In summary, EMG-based reflex activity was consistently observed in sessions where a reflex was independently confirmed via middle ear admittance and electrode placement and integrity were preserved. In contrast, the absence of detectable EMG activity was always associated either with lack of reflex confirmation by admittance measurements or with compromised electrode positioning or connectivity. These findings indicate that, under adequate recording conditions, EMG responses behaved as expected and reliably reflected stapedius reflex activation. Consequently, the observed limitations are primarily attributable to technical and implementation-related factors rather than to the underlying physiological feasibility of EMG-based detection in a chronic setting.

### 3.2. Reflexes Detection

**Figure 3:**
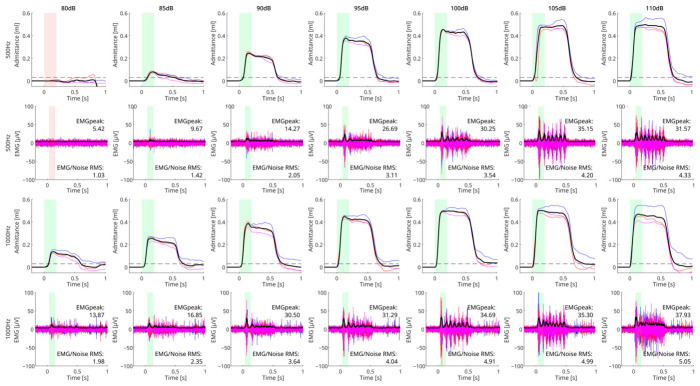
Middle ear admittance (first and third row) and EMG signals (second and fourth row) while stimulation with 500 Hz (the two upper rows) and 1000 Hz (the two lower rows) were presented of sheep S03 during a final measurement with increasing loudness from left to right (80 dB to 110 dB). Each color line represents a repetition and the black line the signal average (for admittance) or average envelope (EMG). The shadow area represents the analyzed area and its color the overall classification, reflex (green) or no reflex (red).

Overall, SR detection via middle ear admittance corresponded with the presence of the EMG with both signals synchronized to the stimulus onset (Figure 3).

Figure 3 depicts an example of the recorded signals from a single measurement (Sheep S03), showing the responses to acoustic stimulation compared to the filtered EMG. The third row shows the EMG envelope. As expected, the middle ear admittance signal and EMG increased clearly as the sound level was raised. In a few cases, the EMG signal already showed a reflex that had not yet been detected by the tympanometer device.

Four different patterns could be distinguished in the reflex-related EMG activity: a short burst at the beginning of the stimulus; a short burst followed by smaller tonic activity; then a series of burst spikes; and finally, a tonic activity fusing those spikes. Generally speaking, these four behaviors corresponded to reflexes going from small to very large reflexes.

From the nine sessions in which reflex responses were detected via EMG, a total of 1453 stimuli across all combinations of stimulation intensity and frequency were analyzed. Of these, 915 responses were classified as reflexes based on the predefined criteria. Reflex-related EMG activity exhibited peak amplitudes ranging from 3 to 68 µV RMS, with a mean (±SD) of 20.2 ± 12.3 µV RMS. The corresponding RMS ratios ranged from 1 to 12.3, with a mean (±SD) of 3.8 ± 3.1.

The classification procedure showed a high level of internal consistency, with only 33 out of 1453 stimuli (~2.4%) requiring reclassification during visual review (17 from no reflex to reflex and 18 from reflex to no reflex), indicating that the quantitative criteria provided a stable initial classification despite variability in recording conditions.

Due to the low number of animals, statistical analysis is not possible as samples would be diluted among all variables. However, descriptive statistics per session are provided in Supplementary Table S1.

### 3.3. Histology

During implantation, it was noticed that the facial nerve and the stapedius muscle were not enclosed separately by bone as in humans but were located together in the sinus tympani. Only a thick layer of connective tissue isolated the two structures from each other. In addition, the stapedius muscle was bulkier than in humans, in which it is relatively slender and long (Figure 2D). The stapedius could be reached in every animal via the retrofacial approach, as it always became visible during drilling at the very lateral aspect of the geniculum of the facial canal. The labyrinth was in some cases very close to the muscle (0.5 mm). It was accidently injured in two animals (Figure 2C). The animals recovered within a few days, and the experiment was continued as planned. Access via the middle ear, on the other hand, would be much more complicated in sheep, as the tendon of the muscle disappeared under the facial nerve and no pyramidal eminence is developed (Figure 2C).

**Figure 4:**
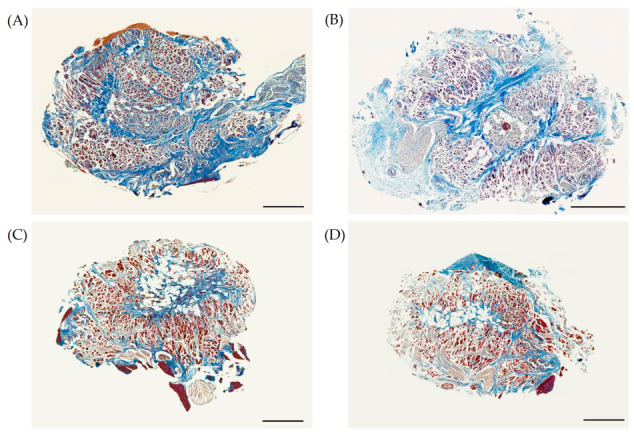
Histological comparison between the left (implanted) and right (non-implanted) stapedius muscles. Sections were stained with AZAN by Heidenhain (connective tissue in blue, muscle fibers in red, nerve branches in light red, fat cells colorless, bone in red with blue spots), scale bar = 500 µm. (**A**) section of the left implanted stapedius muscle of S06 at the electrode position; (**B**) section through a corresponding area of the right non-implanted stapedius muscle of S06; (**C**) section of the left stapedius muscle of S04 (sham-implanted animal); (**D**) corresponding section of the non-implanted right stapedius muscle of S04.

No differences were found between the left and right stapedius muscle of the two controls (S04 and cadaveric control) or in the implanted animals. No signs of atrophy or fibrosis were evident in any of the left stapedius muscles. In addition, no scar tissue or noticeable accumulations of fat were detected despite the implantation of EMG electrodes (Figure 4). However, the proportion of fat present at the proximal end of the intramuscular tendon, distal to the stapes head, varied across animals. Both independent raters identified sheep S05 as having the greatest amount of intramuscular fat within both stapedii. Nevertheless, the stapedius reflex was successfully elicited during pre-screening.

Since the electrodes were placed on the surface of the muscle rather than inside, the implantation did not appear to cause any damage to the muscle tissue or noticeable scarring. Thus, the electrodes could be removed without any problems after 6 months. Even the detachment of the muscle fibers from the wall of the cavity to prepare the pocket did not lead to any visible muscular changes.

**Figure 5:**
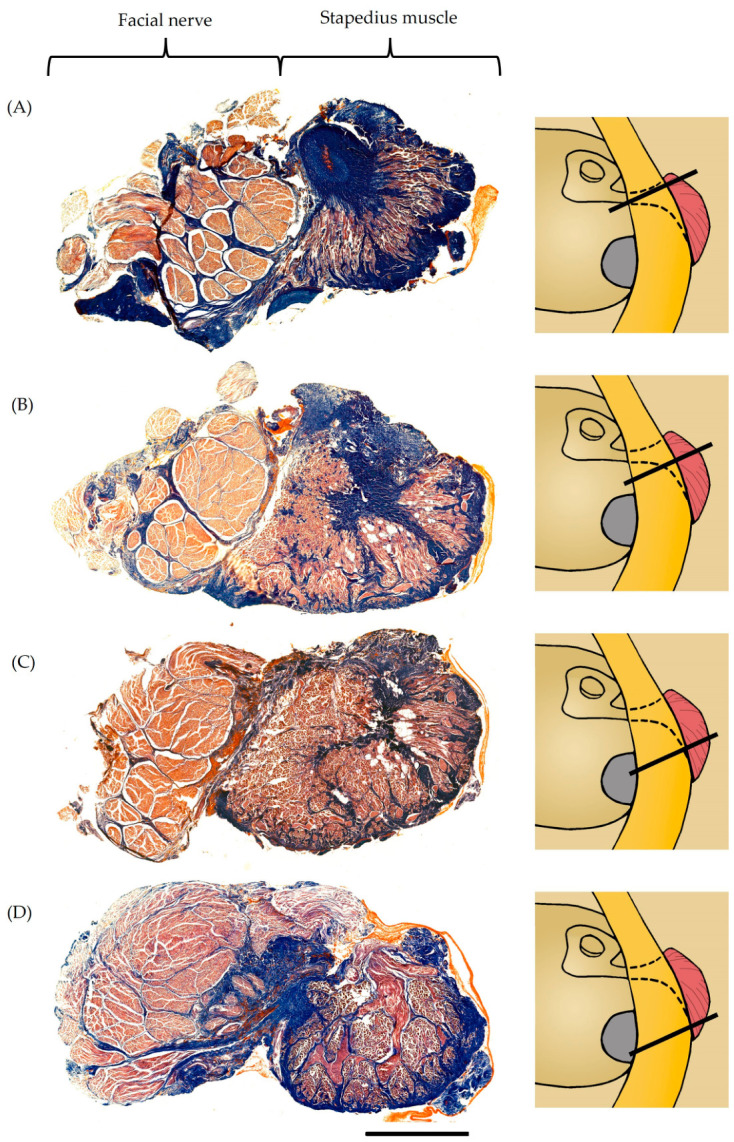
Partitioning of a non-implanted stapedius muscle of the control (AZAN staining, muscle fibers in red, nerve branches in orange, connective tissue in blue, fat cells colorless), scale bar 1 mm. (**A**) close to the stapes, the stapedius tendon is prominent and a part of the bony insertion (spike of the stapes head) is visible; (**B**) in the middle region of the muscle, the tendon is surrounded by muscle fibers; (**C**) in the proximal third the muscle fibers dominate, the tendon is nearly gone and accumulation of fat cells are noticeable at tendons origins; (**D**) the surface of the proximal region is characterized by many nerve branches.

However, all examined stapedius muscles could be divided into three morphologically distinct regions: a distal, a middle, and a proximal region (Figure 5). Each region was characterized by a specific composition.

Distally, i.e., close to the stapes, the intramuscular tendon was very prominent in all animals and occupied a large area of a cross-section. The few muscle fibers were longitudinally aligned in the cross-sections and spanned between the wall of the cavity and the tendon (Figure 5A). In the middle region, the tendon lost its compactness and spread across the entire cross-section (Figure 5B). It remained prominent but was now almost completely surrounded by muscle fibers. On the side facing the facial nerve, the muscle fibers ran transversely, and on the opposite side continued to run longitudinally. The tendon was also surrounded by some fat cells. Superficially, near the bone wall, thicker nerve branches followed the course of the stapedius muscle. Proximally, the muscle fibers clearly predominated (Figure 5C). The tendon receded into the background but was still surrounded by fat cells. The majority of the muscle fibers, approximately 2/3, were oriented transversely. The rest attached to the tendon at a 90° angle. The longitudinally oriented fibers were found throughout the stapedius muscle on the side facing away from the facial nerve. In the proximal region, close to the wall of the cavity of the petrous bone the innervating nerve branches were located. It seemed, that they formed a kind of network on the surface of the stapedius muscle (Figure 5D).

### 3.4. Adverse Events

In two sheep (S03, S10), the vestibular system was damaged during the implantation surgery. The severity of the damage varied from slight to problematic for 3 days post-implantation. In all cases the sheep recovered.

In another case, the skin incision had been infected due to poor adaptation of the wound edges intraoperatively (S07) and another sheep had skin healing problems around the connector (S04). Both issues had no serious consequences and the animals recovered within one to three days.

## 4. Discussion

The results of this study demonstrate that chronically implanted EMG electrodes can reliably detect stapedius reflex activity in sheep after acoustic stimulation over a six-month period, provided that electrode integrity and correct placement are maintained. This confirms the feasibility of long-term electromyographic monitoring of the stapedius muscle and supports the potential for integrating such electrodes into CI systems to enable objective fitting. The consistency between EMG and middle ear admittance signals, particularly the graded increase in response amplitude with increasing stimulus intensity, reinforces the validity of EMG-based detection. Moreover, the classification metrics used—peak amplitude and RMS ratio—were robust, with only minor manual corrections required during visual screening.

Although middle ear admittance measurements provided a useful comparative reference, their reliability was limited under the experimental conditions. Variability in probe positioning and recording stability introduced uncertainty in reflex detection, which must be considered when interpreting agreement between EMG- and admittance-based outcomes. Importantly, discrepancies between the two modalities do not necessarily indicate incorrect EMG classification but may instead reflect limitations of the admittance recordings. In this context, EMG offers a direct electrophysiological measure of stapedius muscle activation that is independent of middle ear mechanical conditions and may therefore provide a more robust indicator of reflex activity when admittance measurements are compromised. When comparing all objective methods for determining the MCL, the best correlation was found for postoperative eSRT [7,11,18,22,49], which is therefore ideal for CI fitting. However, clinical application remains limited (<39%) [31,40] because additional equipment (impedance meter) is required, which must be operated by an experienced audiologist and the structures between the eardrum and the stapedius muscle must be intact. In addition, the results can be affected due to fluid in the middle ear, the condition of the eardrum, inflammation or problems within the ossicle chain, and, above all, movement by the patient during the measurements [30,50]. This makes the objective determination of the reflex threshold inaccurate and time-consuming, especially with young children or patients with disabilities [41]. Our use of long-term implanted EMG electrodes addresses many of these limitations by bypassing the middle ear and enabling direct measurement of stapedius muscle activity without additional equipment during CI fitting, if this method is implemented within CI technology. This could facilitate the work of the audiologists, improve the quality of patient care, and stapedius reflex related research studies.

In contrast to Pitt et al. (2021) [42], several studies have demonstrated changes in both eSRT and MCL, particularly during the first year after CI implantation [7,12,13,23,31,36,37,38,39,51,52]. Observed changes in neural response telemetry (NRI) or electrically evoked compound action potentials (ECAP) in the first postoperative year support these findings [12,33]. Additionally, further studies have shown that even small changes can affect subsequent speech recognition [37,41]. Consequently, we assume that patients would benefit from regular and accurate determination of eSRT [51] and the associated fitting of the implant. The reason Pitt et al. (2021) were unable to detect any changes in eSRT over time [42], was likely due to their recruitment of patients who, on average, already had 56 months of CI experience. However, the strongest increases in eSRT occur within 6 months and further changes are completed within a year [7,41]. This can be attributed to the ingrowth of the CI electrodes and the corresponding scarring [30,50]. However, as CIs are current-controlled devices that deliver a constant current regardless of the surrounding tissue, it is likely that the changes are primarily due to neuroplasticity—i.e., the brain’s adaptation to new stimuli.

To reliably record the myoelectrical signals of the stapedius muscle for eSRT measurements, correct electrode placement is essential. Our histological analysis showed no evidence of atrophy, fibrosis, or scarring in the implanted stapedii. This finding suggests that surface electrode placement via the retrofacial approach [46,47] at the muscle belly does not compromise muscle integrity, even after six months. This is a crucial observation, as the stapedius muscle is a small and delicate structure, containing on average only about 400 muscle fibers [53], and measuring approximately 2 mm in diameter and 5 mm in length. Thus, any intramuscular damage could easily impair its reflex function. Based on the findings of Walluks et al. (2024), who reported the formation of a 500 µm thick connective tissue ring around an intramuscular implanted electrode [54], a complete loss of the stapedius muscle would be expected after this kind of implantation. Nevertheless, Zarowski et al. (2021) demonstrated successful stapedius reflex measurements by a middle ear admittance following the chronic implantation of an EMG electrode [29]. In this study, EMG measurements were performed exclusively intraoperatively in humans. Postoperatively, the researchers monitored over a six-month period whether the stapedius reflex could still be elicited. Since the monopolar electrode they used was positioned at the pyramidal eminence, i.e., in the distal region of the muscle with fewer muscle fibers and a prominent tendon, this may have prevented damage to the actual proximal muscle belly and led to the positive results. Nevertheless, the EMG signal is the summation of action potentials of many muscle fibers located within the detection range of the electrodes. In our case, all recorded muscle fibers were located between the two recording electrodes, as they were positioned on the muscle surface (Figure 2E). For placement, the proximal portion—that is, the actual muscle belly—was selected to capture the activity of as many muscle fibers as possible. The distal part, where the tendon is prominent, would be less suitable for an EMG recording due to the limited number of muscle fibers.

In two animals, the labyrinth was damaged during exposure of the muscle, leading to balance problems. It can be assumed that such damage is avoidable in human surgeries, as the anatomy of the sheep differs slightly from that of humans in this regard. In humans, the muscle is longer and slender, lies in a separate cavity, and its muscle belly is therefore slightly farther away from the labyrinth. Arnold et al. 2022 & 2026 and Guntinas et al. 2022 demonstrated that the retrofacial approach can be used in humans to place electrodes on the stapedius muscle safely [46,47,55]. In addition, the surgical technique differed. Thus, in contrast to human cochlear implantation, the middle ear was left intact, enabling the reflex to be elicited acoustically as planned. The stapes and the direction of the tendon could therefore not be used for orientation, which complicates the exposure of the muscle. Additionally, the mastoid process in sheep is more compact and the proximity to the labyrinth cannot be determined by the end of pneumatization. With the aid of a preoperative CT scan and the opened middle ear, the risk of injury to the vestibular organ should be low in human surgeries.

The reflex absence in some animals despite histological evidence of the integrity of the treated stapedius muscle, intact electrodes and preoperative reflex confirmation highlights the sensitivity of the stapedius reflex to anesthesia and surgical manipulation [29]. The disappearance of the reflex could be attributed to a stapedius nerve damage, which enters the muscle at the implantation side. However, the nerve regeneration growth rate of 1 mm per day [56,57] and the continuous co-contraction of the tensor tympani muscle [53] argue against such an injury and instead support the notion of a reflex that cannot, in fact, be elicited. It is just speculative, but the tensor tympani muscle is innervated by a nerve branch of the mandibular nerve, which cannot be affected by surgical procedure. Its contractions should still be measurable by middle ear admittance, and the stapedius muscle is expected to be reinnervated within a month. We therefore assume that the use of isoflurane, which is known to suppress reflex responses [58], likely contributed to the intraoperative loss of reflexes in several cases. We attribute the observed difference—namely that the reflex could be elicited in some animals but not in others under the same anesthetic protocol—to individual variability in sensitivity. Inconsistent responses to anesthesia protocols were also observed in human patients [59]. Due to this problem, we adjusted the anesthesia regimen in subsequent experiments so isoflurane could be completely omitted during the measurement phases. This led to a significantly improved ability to elicit the reflex. Furthermore, an elevated stapedius reflex threshold during surgery has been described in several papers [58,60,61]. Since the volume of the eTymp is limited to 110 dB for safety reasons, this could also prevent the reflex from being elicited in some animals.

Technical challenges such as signal variability due to cable shielding and connector stability were consistent with the general knowledge but are expected to be mitigated in clinical CI systems through integrated, miniaturized hardware. Importantly, these limitations did not preclude reflex detection in animals with intact electrodes and preserved reflex pathways.

### Limitations

Although the ovine model offers anatomical similarities to the human temporal bone [45], key differences—such as the absence of the pyramidal eminence and the shared cavity of the stapedius muscle and facial nerve—limit the direct translatability of the surgical approach. These anatomical constraints may not fully reflect the clinical conditions during human cochlear implant (CI) surgeries, particularly regarding electrode placement and access to the stapedius muscle. However, Arnold et al. 2022 and Guntinas et al. 2022 demonstrated that access to the stapedius muscle via the retrofacial approach is feasible also in humans [46,47].

The present study showed progressive loss of electrode and lead integrity over time. This issue primarily affected the continuity of EMG recordings rather than the physiological elicitation of the stapedius reflex itself. The study was designed to assess whether chronic implantation compromises stapedius muscle function or reflex excitability; importantly, reflex responses remained detectable months after implantation when electrodes were functional, and histology demonstrated preserved muscle integrity.

The observed lead failures are likely influenced by the experimental configuration, including cabling fixation to facilitate surgical re-entry, and the mechanical demands of a freely moving ovine model. These factors differ substantially from the fully implantable, strain-relieved lead systems used in clinical cochlear implants, which have demonstrated long-term mechanical robustness. Accordingly, while electrode breakage represents a relevant feasibility constraint in this preclinical setup, it should not be interpreted as a direct predictor of long-term reliability in a clinical implementation.

Another limitation of this study lies in the exploratory nature of the EMG classification approach. As no prior reports of chronic stapedius EMG recordings exist, threshold selection was based on empirical characterization of the acquired data and was not validated using an independent dataset. In addition, the use of a secondary visual review, although guided by physiological criteria such as reproducibility and intensity dependence, introduces potential observer bias. This bias, however, is limited by the small amount of re-classified data (~2.3%).

The use of acoustic stimulation in this study, while enabling the acquisition of EMG signals free of electrical interference, does not fully replicate the conditions under which eSRT are typically measured in cochlear implant users. Electrical stimulation introduces substantial artefacts in EMG recordings due to the proximity of the stimulation source to the stapedius muscle, which can obscure low-amplitude physiological responses [25,26,27,28,29]. However, in acute human intraoperative settings, we have demonstrated that such artefacts can be mitigated through dedicated signal processing techniques, enabling reliable extraction of stapedius EMG activity even during CI stimulation [62]. Therefore, while the present study focused on establishing the physiological viability of long-term stapedius EMG recordings under controlled conditions, future work will need to integrate and validate robust artefact suppression strategies to enable translation to CI-based applications.

## 5. Conclusions

This study demonstrates that the stapedius reflex remains physiologically elicitable following long-term implantation, and that chronic placement of surface EMG electrodes does not compromise stapedius muscle integrity. Reflex-related EMG responses were observed months after implantation in animals with elicitable reflexes, and histological analysis confirmed the absence of muscle atrophy, fibrosis, or chronic inflammatory changes. Together, these findings support the preservation of both the anatomical and electrophysiological properties of the stapedius muscle in a chronic implantation setting.

The use of acoustic stimulation allowed for the characterization of stapedius EMG responses under controlled conditions, free from electrical interference. However, this does not fully reflect the environment of cochlear implant-based eSRT measurements, where stimulation artefacts represent a significant challenge. Integration of artefact suppression techniques will therefore be essential for clinical translation.

While electrode degradation limited longitudinal data continuity in this experimental setup, the preservation of reflex excitability at the six-month endpoint indicates that chronic implantation itself does not abolish stapedius reflex function. These results provide a physiological foundation for future development of EMG-based stapedius reflex monitoring. Further work is required to improve recording robustness, validate artefact-resistant acquisition strategies under electrical stimulation, and assess applicability in human subjects.

## Figures and Tables

**Table 1:** Experimental flowchart and outcome classification. Following implantation, the first follow-up measurement was conducted at 1 month. The second follow-up, scheduled for 3 months after implantation, could not be performed due to electrode-related issues. Nevertheless, it was possible to connect the electrodes proximal to the breakages in all animals to perform the final measurements at 6 months. Green = successful detection of the stapedius reflex; orange = no stapedius reflex detectable; grey = skipped/not measured. The numbers 1 and 2 signed one or two broken electrodes; “-” = no sample excised; “X” = treated sample excised; “*” = untreated control sample.

	Implantation			Follow-Up 1			Final Measurement			Histology	
Sheep	eTymp Pre-Screen	eTymp Intra- Operative	EMG Intra- Operative	eTymp Pre-Screen	eTymp Intra- Operative	EMG Intra- Operative	eTymp Pre-Screen	eTymp Intra- Operative	EMG Intra- Operative	Left Stapedius	Right Stapedius
S03						1				-	*
S04						1				*	*
S05						2				X	*
S06						2				X	*
S07						2				X	*
S08										-	-
S09						2				X	*
S10						2				X	*
S11						2				X	*
Control										*	*

## Data Availability

The data that support the findings of this study are available from the corresponding author upon reasonable request.

## Supplementary Materials

The following supporting information can be downloaded at: https://www.mdpi.com/article/10.3390/s26134224/s1, Table S1: Descriptive statistics of the EMG reflex characteristics across all sessions.

## Abbreviations

The following abbreviations are used in this manuscript:

CI	cochlea implant
DH	drilled hole
EABR	electrically evoked auditory brainstem responses
ECAP	electrically evoked compound action potentials
EMG	electromyography
eSRT	electrically evoked stapedius reflex threshold
FN	facial nerve
HM	handle of the malleus
I	incus
MCL	maximum comfortable loudness level
MP	mastoid process
NRT	neural response telemetry
OEC	outer ear channel
PM	promontory
RW	round window
SM	stapedius muscle
SPL	sound pressure level
SRT	stapedius reflex threshold
ST	stapes
TM	tympanic membrane
TMA	area of the temporalis muscle
UKJ	University Hospital Jena
VO	vestibular organ
