# Supplementary material for: Long-Term Electromyographic Monitoring of the Stapedius Reflex via Implanted Electrodes in Sheep: Toward Objective Autonomous Cochlear Implant Fitting
Source: Sensors (Basel). 2026 Jul 3;26(13):4224. doi: 10.3390/s26134224 (PMC13364023; doi:10.3390/s26134224)
Supplement: Supplementary file 1 [file sensors-26-04224-s001.zip › sensors-4334300-supplementary.pdf]

Supplementary Table 1: Descriptive statistics of the EMG reflex characteristics across all sessions

Subject	Session	Stimuli n	Reflexes n	EMG-Peak Mean (μV_{RMS})	EMG-Peak SD (μV_{RMS})	EMG-Ratio Mean	EMG-Ratio SD
S03	IMP	30	29	12.76	3.20	2.66	0.70
S03	1FU	238	182	14.25	4.29	1.51	0.29
S03	TER	142	131	29.33	8.58	2.83	1.28
S06	IMP	107	58	8.20	3.02	1.95	0.62
S06	TER	132	79	18.72	5.46	1.85	0.54
S07	IMP	50	17	10.55	1.84	1.32	0.15
S07	TER	527	361	24.57	15.12	6.68	3.14
S09	IMP	209	46	10.89	2.40	1.31	0.17
S09	TER	18	12	16.62	2.89	1.31	0.15
TOTAL		1453	915	20.23	12.32	3.82	3.12
